# Supplementary material for: Intersexual Differences in Behaviour and Resource Use of Specialist Phengaris teleius Butterflies
Source: Insects. 2022 Mar 6;13(3):262. doi: 10.3390/insects13030262 (PMC8951063; doi:10.3390/insects13030262)
Supplement: Supplementary file 1 [file insects-13-00262-s001.zip › Supplementary Material S2.pdf]

insects MDPI

Supplementary Material 2

Inter-sexual differences in behaviour and resource use of specialist *Phengaris teleius* butterflies

Miloš Popović [1,2], Ana Golubović [3] and Piotr Nowicki [1]

[1] Institute of Environmental Sciences, Jagiellonian University, Gronostajowa 7, 30-387 Kraków, Poland
[2] Department of Biology and Ecology, Faculty of Sciences and Mathematics, University of Niš, Višegradska 33, 18000 Niš, Serbia
[3] Institute of Zoology, Faculty of Biology, University of Belgrade, Studentski trg 16, 11000 Belgrade, Serbia

1. Butterfly Tracking Study

1.1. Average Flight Time

Table S1. Average time spent on flying recorded during the butterfly tracking study. Original model contained three explanatory variables: sex (females and males), locality (Selevenj and Ludaš) and mowing regime (mown and unmown medow).

Parameter estimates	Estimate ± SE	Z value	P value
Intercept	2.307 ± 0.150	15.352	**< 0.001**
Sex (male)	0.577 ± 0.194	2.979	**0.003**
**Estimates of response**	**Rate ± SE**		
Females	10.0 ± 1.5		
Males	17.9 ± 2.2		
**Contrasts**	**Ratio ± SE**	**Z ratio**	**P value**
Females / Males	0.561 ± 0.109	-2.979	**0.003**

Statistically significant *P* values are shown in bold. Variance of the individual variation (random effect) was 0.636 ± 0.798 based on 330 observations of 65 individuals. Poisson mixed model was fitted using *lme4*, while estimates of response and contrasts are calculated in *emmeans* package in R. AIC of the model was 4844.5. Other supported models include the sex + locality (AIC = 4844.8) and sex * locality (AIC = 4845.0).

1.2. Average Interaction Time

Table S2. Average time spent on interactions during the butterfly tracking study. Original model contained two explanatory variables: sex (females and males) and locality (Selevenj and Ludaš).

Parameter estimates	Estimate ± SE	Z vlaue	*P* value
Intercept	2.252 ± 0.224	10.036	**< 0.001**
Sex (male)	-0.780 ± 0.284	-2.743	**0.006**
**Estimates of response**	**Rate ± SE**		
Females	9.51 ± 2.14		
Males	4.36 ± 0.77		
**Contrasts**	**Ratio ± SE**	**Z ratio**	***P* value**
Females / Males	2.18 ± 0.62	2.734	**0.006**

Statistically significant *P* values are shown in bold. Variance of the individual variation (random effect) was 0.765 ± 0.875 based on 100 observations of 46 individuals. Poisson mixed model was fitted using *lme4*, while estimates of response and contrasts are calculated in *emmeans* package in R. AIC of the best model was 992.4 without support for more complex models.

Table S3. Average time spent on interactions during the butterfly tracking study. Original model included significant variables from Table S2 and an additional variable to diferentiate interspecific (different species) and intraspecific (same species) interactions.

Parameter estimates	Estimate ± SE	Z vlaue	*P* value
Intercept	0.582 ± 0.309	1.887	0.059
Interaction (intraspecific)	1.303 ± 0.285	4.577	**< 0.001**

Estimates of response	Rate ± SE		
Interspecific interactions	1.79 ± 0.55		
Intraspecific interactions	6.59 ± 1.12		

Contrast	Ratio ± SE	Z ratio	*P* value
Interspecific interactions / Intraspecific interactions	0.272 ± 0.077	-4.577	**< 0.001**

Statistically significant *P* values are shown in bold. Variance of the individual variation (random effect) was 0.960 ± 0.980 based on 76 observations of 39 individuals. Poisson mixed model was fitted using *lme4*, while estimates of response and contrasts are calculated in *emmeans* package in R. AIC value for the model was 678.7.

Table S4. Comparison of average interspecific interaction times for individuals of the same sexes in contrast to individuals of different sexes.

Parameter estimates	Estimate ± SE	Z vlaue	*P* value
Intercept	2.401 ± 0.202	11.859	**< 0.001**
Same sex	-1.109 ± 0.198	-5.594	**< 0.001**

Estimates	Rate ± SE		
Different sex	11.03 ± 2.23		
Same sex	3.64 ± 0.78		

Contrast	Ratio ± SE	Z ratio	*P* value
Different sex / Same sex	3.03 ± 0.60	5.594	**< 0.001**

Statistically significant *P* values are shown in bold. Variance of the individual variation (random effect) was 0.944 ± 0.972 based on 58 observations of 32 individuals. Poisson mixed model was fitted using *lme4*, while estimates of response and contrasts are calculated in *emmeans* package in R. AIC value for the model was 562.2.

1.3. Average Feeding Time

Table S5. Average time that *Phengaris teleius* butterflies spent on feeding during the tracking study. Original model contained three explanatory variables: plant (*Sanguisorba officinalis* and other Asteraceae plants), sex (females and males) and locality (Selevenj and Ludaš).

Parameter estimates	Estimate	Z value	*P* value
Intercept	2.904 ± 0.195	14.922	**< 0.001**
Plant (Asteraceae)	1.466 ± 0.245	5.978	**< 0.001**
Sex (male)	-0.382 ± 0.285	-1.338	0.181
Interaction term (Asteraceae × male)	-0.448 ± 0.226	-1.981	0.055

Estimates of response	Rate ± SE
Females on *S. officinalis*	18.2 ± 3.55
Females on Asteraceae plants	79.0 ± 21.14
Males on *S. officinalis*	12.5 ± 2.61
Males on Asteraceae plants	31.4 ± 6.59

Contrasts	Ratio ± SE	Z ratio	*P* value
Females on *S. officinalis* / Female on Asteraceae	0.231 ± 0.056	-5.978	**< 0.001**
Females on *S. officinalis* / Males on *S. officinalis*	1.465 ± 0.418	1.338	0.538
Females on *S. officinalis* / Males on Asteraceae	0.581 ± 0.166	-1.906	0.226
Females on Asteraceae / Males on *S. officinalis*	6.346 ± 2.163	5.421	**< 0.001**
Females on Asteraceae / Males on Asteraceae	2.516 ± 0.860	2.698	**0.035**
Males on *S. officinalis* / Males on Asteraceae	0.396 ± 0.054	-6.809	**< 0.001**

Statistically significant *P* values are shown in bold. Variance of the individual variation (random effect) was 0.739 ± 0.859 based on 116 observation of 42 individuals. Poisson mixed model was fitted using *lme4*, while estimates of response and contrasts are calculated in *emmeans* package in R. AIC of the best model was 1661.8. Other supported models included plant * locality + sex (AIC = 1663.4), plant + sex (AIC = 1663.6) and plant * sex + locality (AIC = 1663.8).

1.4. Average Resting Time

Table S6. Average time spent on resting during the tracking study. Original model contained two explanatory variables: sex (females and males) and locality (Selevenj and Ludaš).

Parameter estimates	Estimate ± SE	Z value	*P* value
Intercept	1.819 ± 0.435	4.196	**< 0.001**
Sex (male)	-0.648 ± 0.680	-0.952	0.341
Locality (Selevenj)	-0.129 ± 0.513	-0.252	0.801
Interaction term (male × Selevenj)	1.482 ± 0.822	1.802	0.072

Estimates of response	Rate ± SE
Females at Ludaš Lake	6.16 ± 2.67
Females at Selevenj Sand	5.42 ± 1.49
Males at Ludaš Lake	3.23 ± 1.69
Males at Selevenj Sand	12.47 ± 4.63

Contrasts	Ratio ± SE	Z ratio	*P* value
Females at Ludaš / Females at Selevenj	1.138 ± 0.584	0.252	0.994
Females at Ludaš / Males at Ludaš	1.911 ± 1.300	0.952	0.777
Females at Ludaš / Males at Selevenj	0.494 ± 0.282	-1.235	0.605
Females at Selevenj / Males at Ludaš	1.679 ± 0.995	0.875	0.818
Females at Selevenj / Males at Selevenj	0.434 ± 0.201	-1.804	0.271
Males at Ludaš / Males at Selevenj	0.259 ± 0.166	-2.104	0.152

Statistically significant *P* values are shown in bold. Variance of the individual variation (random effect) was 1.192 ± 1.092 based on 111 observations of 38 individuals. Poisson mixed model was fitted using *lme4*, while estimates of response and contrasts are calculated in *emmeans* package in R. AIC of the selected model and model that included locality as a sole predictor was 1485.5. Other supported model include sex (AIC = 1485.8) and sex + locality (AIC = 1486.6).

2. Mark Release Recapture Study

2.1. Intersexual Differences in Plant Preferences

Table S7. Intersexual differences in feeding plants of *Phengaris teleius* grouped in three classes based on phylogeny and sample size.

Parameter estimates	Estimate ± SE	Z value	*P* value
Asteraceae	0.308 ± 0.219	1.404	0.160
Other plants	0.693 ± 0.327	2.118	**0.034**
Sanguisorba officinalis	-0.383 ± 0.117	-3.265	**0.001**
**Estimates of response**	**Probability ± SE**		
Asteraceae	0.576 ± 0.054		
Other plants	0.667 ± 0.073		
Sanguisorba officinalis	0.405 ± 0.028		
**Contrasts**	**Odds ratio ± SE**	**Z ratio**	***P* value**
Asteraceae / Other plants	0.681 ± 0.268	-0.976	0.592
Asteraceae / *Sanguisorba officinalis*	1.997 ± 0.497	2.778	**0.015**
Other plants / *Sanguisorba officinalis*	2.934 ± 1.020	3.096	**0.006**

Statistically significant *P* values are shown in bold. GLM binomial model was fitted using R base function, while estimates of response and contrasts are calculated in *emmeans* package.

Table S8. Intersexual differences in resting plants of *Phengaris teleius* grouped in four classes based on phylogeny and sample size.

Coefficients	Estimate	Z value	P value
Asteraceae	0.257 ± 0.139	1.854	0.064
Other plants	0.069 ± 0.166	4.150	0.678
Poales	0.376 ± 0.121	3.121	**0.002**
Sanguisorba officinalis	-0.411 ± 0.086	-4.796	**< 0.001**
**Estimates**	**Probability estimate**		
Asteraceae	0.564 ± 0.034		
Other plants	0.517 ± 0.041		
Poales	0.593 ± 0.029		
Sanguisorba officinalis	0.399 ± 0.021		
**Contrasts**	**Odds ration**	**Z ratio**	**P value**
Asteraceae / Other plants	1.207 ± 0.261	8.700	0.821
Asteraceae / Poales	0.888 ± 0.163	-6.470	0.917
Asteraceae / *Sanguisorba officinalis*	1.952 ± 0.318	4.098	**< 0.001**
Other plants / Poales	0.735 ± 0.151	-1.497	0.439
Other plants / *Sanguisorba officinalis*	1.617 ± 0.302	2.568	**0.050**
Poales / *Sanguisorba officinalis*	2.198 ± 0.325	5.323	**< 0.001**

Statistically significant *P* values are shown in bold. GLM binomial model was fitted using R base function, while estimates of response and contrasts are calculated in *emmeans* package.

3. Overview of the Plant Usage

Table S9. The list of plant species and other objects used by *Phengaris teleius* butterflies for feeding and resting, with percentages of their use shown.

	**Mark-release-recapture**				**Butterfly tracking study**			
	Females		Males		Females		Males	
**Plant/object**	**Feed (%)**	**Rest (%)**	**Feed (%)**	**Rest (%)**	**Feed (%)**	**Rest (%)**	**Feed (%)**	**Rest (%)**
Sanguisorba officinalis	77.83	53.90	61.31	36.78	90.11	70.43	43.66	40.48
Serratula tinctoria	14.78	7.95	18.59	11.00	8.79	11.30	43.66	52.38
Epilobium sp.	3.39	0.48	3.52	2.30				
Knautia integrifolia	0.43		4.02	0.33				
Centaurea jacea	0.43	4.61	2.51	6.08		3.48	12.68	
Stenactis annua	0.43	0.79	2.51	0.66				
Viccia cracca	0.43	0.16	2.01	0.16				
Trifolium pratensis			1.51	0.16				
Fabaceae	0.43		0.50					
Campanula glomerata	0.43		0.50					
Achillea asplenifolia		0.64	1.01	1.31	1.10			4.76
Plantago maritima	0.43	1.91	0.50	2.46		2.61		
Plantago lanceolata			0.50					
Stachis palustris			0.50					
Lathyrus tuberosus			0.50					
Snail shell	0.43	1.59		3.28				
Lotus corniculatus								
Poaceae		8.43		13.63		5.22		2.38
Mollinia sp.		7.15		10.67				
Festuca ovina								
Phragmites communis		1.27		1.15				
Carex sp.		0.16		0.16		0.87		
Genista tinctoria		0.16		0.33				
Galium verum		3.18		3.12				
Equisetum sp.		2.07		1.64		0.87		
Holoschoenus vulgaris		1.27		1.15				
Silene vulgaris		0.64		0.49				
Ononis spinosa		1.75		0.66		0.87		
Linuum perenae/austriacum		0.16						
Cirsium canum				0.16				
Prunella officinalis				0.33				
Other Compositae		0.32						
Cichorium intybus		0.32				2.61		
Salvia sp.		0.16						
Apiaceae		0.16		0.33				
Lepidium cartilagineum								
Medicago sativa								
Dry plant organs		0.64		0.81		1.74		
Sanguisorba officinalis leaf		0.16		0.33				
